# The Experience of Tinnitus and Its Interaction With Unique Life Histories—Life Events, Trauma and Inner Resources Narrated by Patients With Tinnitus

**DOI:** 10.3389/fpsyt.2020.00136

**Published:** 2020-03-18

**Authors:** Soly Inga-Maj Erlandsson, Linda Lundin, Nicolas Dauman

**Affiliations:** ^1^Department of Social and Behavioral Science, University West, Trollhättan, Sweden; ^2^Department of Psychology, CAPS-EA4050, University of Poitiers, Poitiers, France

**Keywords:** tinnitus, self-narration, psychological needs, trauma, frustration, health promotion, sense of coherence

## Abstract

**Background:** The challenges facing people with chronic tinnitus include finding relief and rebuilding quality of life. However, previous traumatic episodes may influence adjustment and prolong suffering. Recovery implies reducing aggravating reactions and improving social roles, relationships and interests. Self-narratives about living with tinnitus have not yet received the attention they deserve in the research literature. Thus, the main goal of the present study was to illustrate how tinnitus suffering interacts with the participants' unique life histories.

**Method:** Four women and one man (ages 52–58) took part in the study after consulting a special hearing clinic for annoying tinnitus. Criteria for inclusion were that tinnitus was regarded as a problem with negative consequences for quality of life. The participants should be willing to share how the experience of tinnitus suffering interacts with their previous life story. Narrative methodology was employed in order to achieve the goals of the study. We used unstructured interviews with free conversation, which allowed for rich narratives with full contextual meaning.

**Results:** The findings, based on the narrative analysis, revealed that three out of five participants presented a *regressive* form of narrative indicating ongoing struggles beyond tinnitus itself, which they were unable to bring to closure. For them, valued goals were continuously thwarted by frustrating circumstances in their lives, either past events or current unresolved issues. *Progressive* and *stable* narratives, as identified in the other two participants, demonstrated values that rely on others' attitude and understanding toward their suffering, in sharp contrast to the *regressive* narratives. We suggest that a central issue in tinnitus rehabilitation should be to help suffering patients to overcome unresolved conflicts and thereby extend their ability for a fuller commitment in life.

**Conclusion:** Considering enduring tinnitus as a chronic condition, whose course is likely to vary depending on the patient's general health status, an alteration of *progressive* and *stable* narratives is likely to occur during the lifespan. A *progressive* narrative shows similarities to the core construct of the salutogenesis model of health promotion (1). In conclusion, a narrative approach in tinnitus rehabilitation can be health promoting by offering the patient the opportunity to engage in storytelling, which in turn can increase comprehensibility and a sense of coherence.

## Introduction

As hearing human beings, we are surrounded by sounds—and sometimes exposed to them—when sounds that we do not want to listen to demand constant attention. For decades, tinnitus obstinately withstood efforts to identify the underlying mechanisms as an element of identifying a cure, i.e., that the patient becomes totally free from the symptoms. But for the one who suffers, tinnitus is an unwanted experienced buzz or tone, perceived in one or both ears or in the head, and which often cannot be ignored. The prevalence of tinnitus in the adult population ranges between 10.1 and 14.5% and it has been reported that 3–4% of adults consult a family doctor about tinnitus at least once in a lifetime (2). Furthermore, approximately 1.6% of the people with tinnitus are considered to be severely annoyed by it (3). It is well-known that the presence of tinnitus cannot solely be explained by the severity of hearing impairment. Although tinnitus can occur in various types of hearing disorders, it can also exist, for example, in relation to brain damage (4), high blood pressure (5), psychological trauma (6), as well as a side effect of certain types of medication (7). Research using experimental methods to investigate the ways in which information is processed on a subconscious and automatic level has revealed that tinnitus sufferers process words related to tinnitus, such as for example “shriek” and “noise,” in a different way than neutral words (8). One possible explanation for this finding is that words associated with tinnitus are experienced as more emotionally charged than neutral for someone who is annoyed by the symptoms. It means that a heightened vigilance can be present for stimuli associated with tinnitus. Indeed, considering that total suppression of tinnitus is an unattainable goal, it is plausible to advocate that patients must learn to cope with persistent frustration (9) that fuels self-consciousness and rumination (10). Rumination may reinforce the amount of frustration of experiencing a condition for which no cure could be found (despite relentless efforts to reduce tinnitus).

The influence of the somatosensory system on tinnitus has long been acknowledged as an important factor to consider in the rehabilitation of patients with tinnitus who complain about myofascial muscle tension [i.e., temporomandibular joint disorder, see for example, (11–13)]. Very little is known about changes in the individual's experience of tinnitus with time, and to what extent and why tinnitus becomes chronic. Wallhäuser-Franke et al. (14) showed that there is a potential risk for the development of chronic complaints in patients who suffer from tinnitus shortly after its onset. On a methodological level, it may be plausible to focus on intra-individual changes through time (i.e., variability) when attempting to search for what shapes and drives a personal experience of the symptoms (15). An alternative to the study of intra-individual variability of tinnitus is the longitudinal study design. Erlandsson and Persson (16) performed a 2-year follow-up of patients complaining of tinnitus, revealing their mental health by the use of psychometry, and psychiatric diagnostic interviews. In some patients, self-rated anxiety and depression were improved at the follow-up; in others the experience of mental problems remained or had increased. In the latter case, reason for the increase of experienced mental problems was related to personality disorders.

Difficulties adjusting to a life with tinnitus, such as previous harsh experiences including traumatic episodes, can hamper adjustment and prolong suffering (17).

According to Greenwood (18), a traumatic experience and the feelings associated with this experience might be lost to conscious memory, and thereby cause inhibitions and blockages. Although the memory of the traumatic incident is blocked, the feelings can still be triggered by a similar experience, as for example in the form of a tone, a voice or a place. In a case study from a psychodynamic, narrative perspective, Dauman and Erlandsson (19) illustrated the complex relationship between sensory fragments (i.e., auditory hallucinations and tinnitus) and a non-disclosed trauma, which occurred half a century earlier in the patient's life. Using a chart review, Fagelson (20) investigated the association between tinnitus annoyance and post-traumatic stress disorder (PTSD) in a sample of 300 veterans who were receiving treatment for tinnitus. Around 34% of the study group carried a diagnosis of PTSD. Almost all of those reporting tinnitus and PTSD announced that hearing tinnitus reminded them of traumatic experiences leading to anxiety, which also seemed to increase the perceived tinnitus loudness.

In contrast to traumatic memories, personal narratives necessitate a temporal sequencing and order (21). This observation may lead to a broader perspective on circumstances of life that have the power to disrupt life existence. Difficulties creating a narrative may well-reflect the individual's disorganization as a result of a traumatic encounter. Thus, for the one who suffers from unprocessed and unintegrated trauma, tinnitus can create chaos, agony, demand attention, constant adjustment, and vigilance. Crossley (22) pointed out the extent to which traumatic experiences, such as chronic illness, threaten the individual's orderly sense of existence and coherence. She further argued that post-traumatic narratives can contribute to the restoration of a sense of order in fragmented self-experiences, following the discursive purpose of building orientation and connections between seemingly unrelated events and emotions. Traumatic experiences can impact an individual's sense of identity and outlook on the future (22). Not only is chronic suffering from the individual's past disruptive, it also outdates previous experiences and orientation in life.

In their review considering trauma etiology and symptoms, Bransford and Blizard (23) stress the likelihood that without knowledge of trauma and its effects, professionals may “misdiagnose individuals and provide treatments that do not address traumatic etiology and that may even exacerbate symptoms” (ibid, p. 87). To recognize signs of a patient's trauma demands the clinician's interest in listening to the person's unheard story. Furthermore, to allow an open dialogue with the patient is the opposite of starting the dialogue from the clinician's own script (24). Here follows an example (unpublished) drawn from psychotherapy with a patient who experienced tinnitus and also suffered from anxiety and depression. Upon beginning psychotherapy, the patient was prescribed with psychotropic medication and had been through treatment of Electroconvulsive therapy (ECT) with no relief. During the course of therapy, clues of an early inhibited trauma became visible mainly through the patient's dreams. The patient's tinnitus and mental problems became unmanageable after retirement and the dreams indicated that leaving work, and particularly work relationships, triggered memories associated with an early trauma. At the age of two, the patient lost his mother to cancer. His father became depressed and unable to care for his children, which led to the patient having to leave his home and live with relatives for some time. At age 14 the patient started to work and stayed at the same company until retirement. Being part of a meaningful social network at work induced a source of secure attachment. After retiring, the patient mourned not only the departure from working life that gave him pleasure and social recognition, but furthermore the patient grieved his dead mother. In Freudian terms, this is an example of “Nachträglichkeit,” i.e., when the patient, 2 years old, is unable to grasp and mourn a severe, traumatic event in the sudden separation from his mother.

The challenges facing people with chronic and severe tinnitus are to retain, or to rebuild, quality of life. With tinnitus, chronic recovery is thus not about eliminating the symptoms; instead it is about reducing the aggravating reactions to the presence of tinnitus, and about recovering social roles, relationships, abilities, possibilities, and interests in daily routines and commitments (9, 25). In personal narratives, individuals can weave together their reconstructed past, perceived present and anticipated future (26, 27). Self-narratives about living with tinnitus have not so far received the attention they deserve in the literature on tinnitus rehabilitation. There are, however, a few studies including patients with tinnitus who have been able to explore self-narratives, for example individual psychotherapy [e.g., Dauman and Erlandsson (19)], group psychotherapy [e.g., Zöger et al., (28)], research interviews [e.g., Dauman et al., (15)] and tinnitus support groups [e.g., Pryce et al., (29)]. They can all be considered attempts aimed at sustaining meaning making as a natural human reaction to the disruption of chronic conditions, thus promoting more progressive narratives among suffering patients (22).

Quantitative research measures provide answers that are detached from context; in the positivistic tradition, the social world is proposed to exist of a concrete and unchangeable reality, which can be quantified objectively. Qualitative research instead focuses on analyzing subjective meaning; in the interpretative tradition, reality is proposed to be socially constructed by humans. Qualitative research is an umbrella term that covers an array of interpretative techniques. The qualitative approach called narrative method further allows the researcher to go beyond the patient's subjective meaning making by collecting a detailed life story that also addresses social, cultural and psychological issues. An individual's experiences in life, relationships, emotions, goals, opinions and cultural values often impact on decisions, behaviors, challenges, well-being, and health. Narratives are frameworks that compose human histories and identities. The human experience is thus aimed to be recognized holistically, where meaning can never be set apart from context. In many theories, human beings are believed to possess a natural and inherent drive to search for meaning and coherence; these factors have great relevance in health research (1). Human beings thus create meaning from their experiences and this meaning making is health promoting and associated with well-being. One of the ways in which a person can search for meaning and coherence is through language. Through self-narratives and by linking various experiences, feelings and reactions, it is possible to create a coherent story about one's life (26, 27, 30). Meaning and coherence are thus created in the act of telling stories, i.e., the narrative creates order from chaos by forming parts of experiences in life into a coherent whole (31). The self-narrative can thus be perceived as a reflection of the search for meaning and coherence, concepts that belong to the Salutogenic model that Antonovsky introduced in his book “Health, Stress, and Coping” (32).

### The Narrative Psychological Approach

Despite the increasingly widespread use of narrative approaches in mainstream psychology, there are very few narrative studies including patients with tinnitus complaints. Narrative research encompasses a *descriptive* and an *explicative* approach (33). In the descriptive approach, narratives are used (from individuals or groups) with the aim to describe and give meanings to conditions of living that should be considered as unique, and therefore beyond comparison with other conditions of living. The individual's subjective experience must be taken into consideration as the condition shapes the whole experience of living and, in turn, how life is narrated. This also means that the narrative disposal—the structure of the interview as well as the interviewer's commitment within it—does contribute to the shape of experience that is individually constructed as a narrative tailored to someone else's understanding. The explicative approach, on the other hand, intends to construct an explanation for *why* a situation/occurrence initiated a human act, i.e., the purpose of the narrative analysis is to reach an understanding of what causes the situation (the final event). In this study, we considered the descriptive approach to be relevant as a first step of analyses, and as a second step we made connections between the narratives and the broader theoretical literature for interpretations of the same (34).

The narrative method is an umbrella for several narrative approaches. One of these methods, i.e., the narrative psychological approach was developed as a way of understanding the psychology of trauma and how people adhere and respond to traumatizing events (22). Although the meaning-making process is important for mental health and well-being, it can nevertheless be difficult for someone with a history of severe trauma to integrate a narrative about life. Narrative psychology implies that the individual can hold a variety of identities and each identity is connected to different social relationships. As the narrator is an active agent and part of a social context, it is possible to understand both the narrators and their worlds (35). Although no one is capable of becoming a complete author of one's life, we can learn to become the narrator of our own story (36).

## AIMS

One of the aims of the present study was to enhance the foundation for making clinical decisions about tinnitus interventions by focusing on the narratives of individuals in need of rehabilitation for tinnitus distress. Based on our clinical understanding, tinnitus is not a phenomenon that simply can be added to the patient's previous experience of life before the symptom begun. This assumption does not consider that tinnitus may also shape the experience of life from top to bottom. To our knowledge, there are few examples in the literature that consider a lay, patient's perspective. The patient's perspective risks being placed in the background. Hence, the study aimed to allow individuals experiencing tinnitus distress to have their voices heard. Following Crossley (22), our intention was to appreciate participants as active social beings formed and influenced by social and cultural norms, who thereby are engaged in a meaning-making process surrounding the onset of tinnitus and its daily consequences. Another part of our main purpose was looking into how the informants attempted to find diverse alternative solutions to their suffering. We were interested in what activities they found helpful, and the kinds of things which possibly made their daily life somewhat more tolerable. Further aspects we intended to explore included the inner resources that the informants could mobilize in order to maintain hopeful about the future.

### Research Questions

What are the challenges that the patients have to face in their daily life, trying to cope with tinnitus?
How does the experience of tinnitus suffering interact with their previous life story?
Has tinnitus contributed to altering their image of themselves? Something they learned about themselves; from others; from a previous good/bad event etc.
In their attempts to find solutions to different stressors in life—What is facilitating? What is hindering?

## Methods

### Participants

The sample comprised four women and one man with tinnitus in the ages of 52–58. The informants had previously consulted a special hearing clinic in Sweden in order to get help for distressing tinnitus and were evaluated for possible hearing impairment (see Table 1). Criteria for inclusion were that they experienced tinnitus as a suffering with somewhat negative consequences for quality of life, and that they were willing to share these experiences with the researchers during three interview occasions over a period of 3 to 4 months.

**Table 1:** Participant demographics.

**Name & gender**	**Age**	**Tinnitus duration**	**Hearing loss duration**
Karin, female	57	2 years	2 years
Ulla, female	55	2 years	2 years
Eva, female	52	10 years	10 years
Frida, female	53	25 years	20 years
Erik, male	58	45 years	13 years

### Interview Protocol

The narrative interviews were conversation-like, where a loosely structured interview guide was followed that encouraged participants to generate detailed accounts of experiences. The aim was to conduct fairly unstructured interviews with free conversation that allowed for rich narratives with full contextual meaning. We set out to achieve exploration of personal narratives by asking questions about participants' backgrounds, for example questions about childhood, family situation, working life, social, and cultural context, etc. We also asked questions about tinnitus, for example how they reacted when they first understood that tinnitus was permanent, how they dealt with their emotional reactions, what type of support they had received, etc. Through these questions, we wanted to gain an inside perspective of each participant's life situation, including previous life circumstances in relation to socio-cultural context. With this information, our aim was to more fully understand reactions and feelings linked to the patients living with tinnitus. The loosely structured interview protocol encouraged participants to talk about any part of life, such as hopes, dreams and fears, etc. Examples of questions appear below:

- Tell me about your experiences living with tinnitus?
- Has the presence of tinnitus made you suffer more than ever before?
- Has your perception of life changed since you had tinnitus?
- Is there anything in particular that has had a significant impact on your life up to now?
- Is there anything you can do to make tinnitus more tolerable?

### Procedure

Participants were recruited from a Deaf- and Hearing Centre in Sweden. Patients who had been evaluated for possible hearing impairment at a special hearing clinic, and who experienced suffering from tinnitus were informed about the research study in writing via informational fliers. If they were interested in participating in the study, the personnel at the Deaf- and Hearing Centre contacted one of the researchers (SIE) and provided contact information for potential participants. The researchers (LL, SIE) phoned those who had expressed interest and booked a time for the first interview, upon agreement to participate in the study. The five participants were interviewed three times and each interview lasted between 40 and 70 min, and took place either at the University, the Deaf- and Hearing Centre, or at the patient's workplace. For one participant who had difficulties traveling to the University, the offer was made to carry out the interviews at the Deaf- and Hearing Centre. All interviews were tape-recorded and transcribed verbatim after each interview session.

### Ethical Considerations

The study was carried out in co-operation with personnel at the Deaf- and Hearing Centre, where the patients were recruited. Clinical preparation and administration required was defrayed by the medical service in agreement with the general manager. The researchers followed the principles of the Declaration of Helsinki [1975; revised (37)], a statement of ethical principles to provide guidance in research involving human subjects, and the study was approved by the Regional Ethical Committee in Sweden, see Protocol No. 32013. Written information about the procedure of the study, including examples of interview questions, was provided to those who decided to participate. It was certified that taking part in the study was voluntary and that they could, without explanation, cancel their participation at any time. Prior to participation they were assured that the study would be anonymous, and that any personal information would remain confidential. They were told that in order to enable a rigorous analysis of their story, the interviews had to be recorded, and that the study results would be published in a scientific journal. The participants gave their written informed consent before the start of the interviews.

### Analysis

Narrative methodology was employed in order to achieve the goals of the study, i.e., a patient perspective on tinnitus suffering and its consequences for quality of life and demands for future life. A transcription was made after each interview in order for the researchers (LL and SE) to reflect and discuss important issues prior to the following interviews. In total, 15 deep, semi-structured interviews (three per participant) were transcribed verbatim by two of the researchers (LL, SE), and together the transcripts amounted to 167 pages. In order for the third researcher (ND, not Swedish spoken) to be involved in the narrative analysis, a major part of the transcripts was translated into English. The first stage of the analysis involved all three researchers reading through the transcripts in order to familiarize themselves with the content. Notes were taken during this process and the researchers' dialogue and analytical input proceeded until they reached the goal of comprehending the content of the narratives, and how these were socio, culturally and contextually positioned. During the process of close reading, a coding frame that captured the links between sub-plots could be applied to each participant's narrative [see Mishler (38)]. The aim of the coding frame was to find the overall meaning of the narratives, while also observing the specific topic in each of those narratives. In a second step, we applied a broader theoretical frame of reference to interpret the participants' stories. This means that we went beyond the descriptive phase, toward the interpretative.

In this study, we used the narrative psychological approaches suggested by Gergen and Gergen (39) and Crossley (22) as those best describe people's experience of negative events and suffering. There are several ways in which we can analyze a story by giving it a structure. According to Sarbin (40), a personal narrative has a temporal dimension (a beginning, a middle and an ending) that is held together by a pattern of events named a plot. Gergen and Gergen (39) classify the plot that people use in making sense of negative events, such as suffering from serious illnesses, into three main structurers: *stability, progression, and regression*. The *progressive* structure is characterized by a movement toward a goal, the *regressive* is defined as mostly a glance backward, and the *stable* as a narrative with little or no change. A classic structure of narrative is Frye's use of the concepts; comedy (i.e., happy ending) romance (somewhat similar to *progressive*), tragedy (to *regressive*) and satire (to *stable*); (41). According to Crossley (22), the tone (i.e., the overall valence of the narrative) should be placed at the center of the narrative. In a *progressive* narrative structure, the tone would be optimistic, while in a *regressive* narrative the tone would be pessimistic. The tone in a *stable* narrative structure would be objective and more like a list of events than a complex story (34).

## Results

The three main structurers of the narratives' pattern of events include *progression* (forward looking), *regression* (backward glancing) and *stable* (little change). As shown in Table 2 below, the narrative structure of Karin, Ulla and Frida was found to be *regressive*, the structure of Eva's narrative is *progressive*, and the structure of Erik's narrative is *stable*. The *tone*, i.e., the overall valence of the narratives, is described as how agency is posited toward events that are narrated and challenging to the participants. The tone of the three participants with *regressive* narratives (Karin, Ulla and Frida) was defined as pessimistic, the tone of Eva's narrative as optimistic, and the tone of Erik's narrative as objective. Recurrent patterns of the participants' intentions are referred to as dominating themes in the narratives (42). Here, the themes, as shown in Table 2, are *struggle* (Karin), *competence* (Ulla), *empathy* (Eva), *sadness* (Frida) and *proudness* (Erik). Excerpts from dialogues of each participant can be found under Supplementary Material.

**Table 2:** The manifestation of the narrative in tone, main structure and theme.

	**Tone**	**Main structure**	**Theme**
Karin	Pessimistic	Regressive	struggle
Ulla	Pessimistic	Regressive	competence
Eva	Optimistic	Progressive	empathy
Frida	Pessimistic	Regressive	sadness
Erik	Objective	Stable	proudness

The tone of **Karin's** narrative is **pessimistic**, although she demonstrates an urge to solve her problems that, besides tinnitus and noise intolerance, are mostly economic at the time of the interviews. She started out trying to fulfill the needs of others but found that this way of life made her vulnerable and ended in a mental breakdown. The structure of her narrative is **regressive** and somewhat **tragic** as she experiences a number of obstacles to stabilizing her life. Trying to drive her own business resulted in failure several times. An uncertain work situation seems to influence her emotional reactions to both loud sounds and tinnitus. As a teacher in music, she experiences a worsening of tinnitus after giving several days of music lessons, also making her more concerned about her hearing. Karin stopped playing the guitar for some time, as tinnitus made her play out of tune. When standing in a gathering with people she has difficulties coping with noise or loud voices. Often it forces her to leave the place, because she doesn't know “what to do.” When tinnitus is exhausting, it often leads to headaches and vertigo. During one interview, Karin brings up memories of a passed long-term depression, a condition that she worries she could experience again. Tinnitus has an impact on her patience, and it becomes more intense when she feels frustrated, however: “it can be managed in one way or another.” The theme in the narrative seems to be a **struggle** to reach what she wishes to obtain for her future life. She maintains her engagement in music activities by playing her own instrument at social events, i.e., birthday parties, weddings, etc. Working in the garden, surrounded by a green environment, Karin has found a way to conquer emotional stress. But she stands by a crossroad where she tries to decide whether to continue working as a teacher. She does not have the legitimate teacher education, and this implies struggling to either maintain a job in an uncertain position or trying to find another position. The latter alternative can be challenging pending on her age.

The tone of **Ulla's** narrative is **pessimistic** because she cannot, with confidence, look forward to a solution to the problem with tinnitus for which she demands help. This situation makes her feel out of control. A traumatic experience of a recent car accident, and the presence of tinnitus, is brought up in a repetitive manner throughout her story. The main structure of the narrative is **regressive** also characterized by **despair**, as she experiences being abandoned and misunderstood by some of the health professionals. Ulla has a university degree—a Master's in Economics, but due to long-term, physical health problems, i.e., scoliosis, she prefers having a flexible work situation and runs a cab business together with her husband. She raises adequate questions regarding the physical consequences of the car accident, but the answers she at times receives from health professionals do not make her calm. One of them has suggested that her tinnitus, which began after the car accident, was stress induced. To think about a life with tinnitus makes Ulla both angry and sad and, in her mind, she dwells about revenge. The general theme in the narrative is **competence** as she sees herself as a skillful person and a qualified cab driver. She used to feel good about her job, which she is able to carry out despite scoliosis. Since the accident, she can no longer join her work mates during lunch hours or at breaks due to sensitivity to sounds, with consequences of her social belonging becoming jeopardized. This is a source of grief. Ulla notices that she no longer can enjoy listening to music in the church, which used to be a way for her to relax, resulting in another reason for her to feel hurt. She looks back at a time in life when she enjoyed being at her previously owned summer house surrounded by nature and silence.

The structure of **Eva's** narrative is **progressive** and her strength and hopefulness toward the future confirm the underlying **optimistic** tone. There are, however, signs of darkness concerning one of her two children whose prospects in life are most uncertain due to chronic illness. Eva believes in her ability to find help along the way and expresses in a moving way how support from her colleagues gives her courage and hope. The theme that seems most prevalent in Eva's narrative is **empathy**. Throughout her story, there are examples of willingness to be caring together with willingness to hold high moral standards. Her colleagues did not know about her tinnitus until some time ago, which is similar to the mother's way of coping with tinnitus: “it is not even worth mentioning” (Eva's citation). Eva grew up in a country close to Sweden with many siblings and a caring mother whom she is in regular contact with. Her identity has a strong connection to her professional life but also to her roots. Eva is proud of her achievements, although her ambitions at times turned into a heavy workload. At the end of the final interview she expresses her appreciation for being able to give a voice to her struggles with the hearing loss and tinnitus, as well as about life in general. Being able to narrate some part of her history was found to be therapeutic and helpful.

The structure of **Frida's** narrative can be characterized as mostly **regressive**, with a **pessimistic** tone. Regardless of a fragile health condition, physical as well as mental, she still manages to work full time within health care for aging people. In addition to hearing loss and tinnitus, Frida has been diagnosed with benign paroxysmal positional vertigo, migraine, asthma and panic attacks. She worries about her eyesight and that her hearing continually declines as she is aging. Disturbing noise from neighbors and in public places contribute to a restricted lifestyle. An unprocessed trauma early in life still tangles with her. As a child she was brutally raped by her father's friend and the abrupt changes that her family had to make moving to another continent were **dramatic**. Although many years have passed, Frida still blames herself for the reason that they left Sweden. A recurring theme of her narrative is **sadness**, and she remains feeling responsible for this irrevocable change of abode. It is enigmatic that Frida is the only one in the family who moved back to Sweden, while her mother, father and siblings remain living on another continent. Frida, like Erik, has a long history of being in physical pain. She knows what it means to be physically restricted, and what it takes to return to a more “normal” form of life. Memories of a suicidal attempt and circumstances that rescued her from the act are still very vivid and painful. When bringing the hurtful incident up during the interview, Frida falls into tears. She is thankful for being able to tell the researcher about her past and regards the narration as a form of therapy.

The tone of **Erik's** narrative is **objective**. His life, since he left work for a sick pension, did not change much, and characteristic for his narrative, he is an individual living in a day-to-day situation that is rather **stable**. However, Erik must be observant of his physical health and moderate his emotions alongside the efforts to stay in a stable position. The central theme in his narrative is **proudness;** Erik is proud of himself—he is a survivor from the “social battlefield.” His narrative is imprinted by a form of **satire** influenced by the story told by those who have made a class journey. His image is more or less built up around this version of his narrative, although he claims not being “God's best child” as a young boy. Erik calls attention to the fact that he is satisfied with his life and what he has accomplished. Other aspects of the narrative are realism and common sense. For Erik, tinnitus is just one health problem among others, of which chronic pain is the most pronounced. For some reason, tinnitus did not become overwhelming as he manages to maintain a tolerant attitude toward its presence. One explanation might be that Erik, from an early age, heard about tinnitus as members of his family were well-acquainted with its presence. Moreover, it was generally seen as a familiar problem for people in a working-class environment.

### Summary of Lasting Effects of the Events on the Narrator's Identity, and How These Events Have Shaped Their Sense of Self When Meeting With Challenges in Life

Having needs of your own has been a sign of selfishness for **Karin**—even in case of illness and emotional hardships. To be a social person with much to offer others has contributed to forming her identity. While not anchored in her own needs, it took time to adjust to the fact that she, like other people, can be fragile and in need of care. This became clear to her in times of suffering as she learned that her self-value cannot depend on fulfilling the needs of others. Caring for herself has been difficult, and therefore the main dimension of Karin's life story is *regressive*. Her strengths (being a caring person) have not been an asset that she could benefit from during difficult periods in life. Instead, she had to be more “selfish” in order to stay healthy.

**Ulla** had to realize that life can be unpredictable, as it was when she was hurt in a car accident that also resulted in the start of her tinnitus. She finds herself in the middle of a crises after the unforeseen event, which occupies her day and sometimes night. There is a considerable impact on Ulla's social life, which used to be part of her enjoyment of working as a cab driver. Ulla's strength is her ability to find new paths in life, and to adjust to a long-term physical condition that has implied several limitations in her way of living. Due to the negative aftermaths of the car accident she is left ruminating about the loss of agency, making her both angry and sad. She continuously experiences frustration as the loss of agency still lingers with her and makes it difficult to move forward in life. Her story, therefore, leans toward a *regressive* pole.

For **Eva**, to be self-sacrificing and not complaining has been a part of her identity since childhood and a mantra through life. Among her strengths is that she is persistent but nevertheless able to acknowledge her limits. Social support gives Eva courage, together with purpose and meaning when she is supportive to others. She is introspective and can look at her life from different horizons. An important asset for Eva is her identification and close relationships with family members in her birth country. Part of her personal weakness is that she can be self-sacrificing with detrimental consequences for her health. Although life circumstances can be tough, Eva is able to appreciate her share in life which points to her narrative as *progressive*. Her self-confidence and strength have helped her to find meaning through social belonging, both familywise and at work.

**Frida** learned that the experience of being secure can be gone in a flash and that your life can change dramatically. She does not convey a sense of belonging in life, instead she conveys loneliness, sadness, guilt, and a sense of alienation. She has carried feelings of shame and guilt about being raped and about feeling responsible for the family's decision to move abroad. Frida experienced many disappointments both as a child and as an adult individual. For example, she grieves not becoming a mother, as motherhood was something she perceived as a natural way to achieve a sense of belonging in many areas in society. Her personal strength is her rich and eventful life, providing her with experience and opportunities to learn how to deal with challenges. Thereby, there is agency even though she is vulnerable due to previous sad experiences. Frida's personal fragility from traumatic incidents has colored her, and she carries unprocessed emotions leading to a life story that is primarily *regressive*.

When struggling with tinnitus and his other health issues, **Erik** is using active problem solving—a strategy that enables him to continue living a life filled with interesting activities, adjusted to his abilities. Erik's story reflects how he, since childhood, has been used to making adjustments in order to overcome disadvantages and hardship, in turn providing him with a strong sense of agency in life. However, he also has to deal with several health problems as he has been physically impaired for a number of years, and his health status may not change for the better. Presumably, his strength has helped him to move forward using mental determination and active strategies for solving problems through life. Those personal traits have contributed to a story line in Erik's narrative that is stable.

## Discussion

The present study belongs to the emerging qualitative research on tinnitus, following earlier works in audiology on hearing impairment (43, 44), Ménière's disease (45), and otosclerosis (46). Starting a decade ago with tinnitus patients (47), the use of qualitative methods enlightening the lived experience of suffering individuals now encompass grounded theory studies (9, 48, 49), IPA studies [i.e., Interpretative Phenomenological Analysis, (50)], thematic analysis studies (51, 52), and mixed methods studies [i.e., qualitative and quantitative, (53)]. Researchers have contributed to extend original open-ended approaches to the patients suffering [e.g., Tyler and Baker (54) and Sanchez and Stephens (55)], furthering the integration of tinnitus into more parsimonious and patient-centered models (9). In a psychodynamic case study, (19) emphasized that narratives of patients suffering from tinnitus are of special clinical value, for the understanding of prior traumatic experiences entangled with tinnitus [see also Fagelson (20)]. The present study extends the scope of this perspective, with a focus on the forms of the narratives (56) told within a research interview setting.

Living with chronic tinnitus may amplify disruptive and alienating conditions that threaten a patient's sense of identity, thereby negating previous routines and self-awareness. In these circumstances, (56) narrative approach is of special relevance to clinicians. Indeed, patients whose narrative testifies for a continuous distancing from any valued goals in life (i.e., *regressive* narratives) should call for the clinicians' attention, in contrast to those showing a possible sense of achievement and agency toward their life goals despite tinnitus hindrance (i.e., *progressive* or *stable* narratives). Three out of five participants in our study were presenting a *regressive* form of narrative (i.e., Frida, Ulla and Karin) indicating ongoing struggles beyond tinnitus itself, which they were unable to bring to closure. For them, valued goals were continuously thwarted by frustrating circumstances in their lives, either past events (Frida) or current, or unresolved issues (Ulla and Karin). A *regressive* form of self-narrative can be related to the perspective of self-determination theory (57), for which a *deprivation* of basic psychological needs leads to the impoverishment and alienation of the self. Conversely, self-determination theory states that the individual's innate tendency toward health, integrity and personal growth is sustained by the fulfillment of such needs throughout the lifespan. Three basic needs—autonomy, competence, and relatedness—have been identified as paving the way for well-being or chronic alienation, depending on whether they are enhanced or thwarted by the social surroundings (57, 58). It is worth noting that the *regressive* narratives in our study give proof of deprivation of these basic psychological needs. The existence of prior trauma (e.g., Frida), ongoing struggle (e.g., Karin), or unattainable goals (e.g., Ulla) shall be taken into account when considering the burden of tinnitus on the life of suffering individuals. An essential aspect of this matter is the amount of resources that are available to the suffering individual *at the onset* of tinnitus.

Stressful events, such as substantial loss, can cause existential questions to come to the fore and pose threats to an individual's conservation of resources (59). In accordance with this perspective, concerns for uncertainty of the future and impoverishment of resources (physical and psychological) are pervasive in patients who are subjected to a chronic condition (60, 61). Thereby, patients who are overwhelmed by facing chronic tinnitus should be considered to be suffering from deprivations and struggles that would have *already* exhausted (and may continue to do so) their inner resources. In addition, it is worth noting that the presence of tinnitus is, in itself, a persistent thwarting of basic psychological needs. Tinnitus can lead to alienation, disablement and isolation (e.g., Ulla) as a result of a repetitive questioning of patients' innate needs for autonomy, competence (i.e., agency), and relatedness to significant others (58). While questionnaires have been the single most used approach in patients with tinnitus, we would like to claim that self-narratives do offer a much deeper understanding of an individual's sense of an orderly existence and available resources to cope with the condition. Thus, approaching the burden of tinnitus as a *regressive* narrative (56) enables clinicians to consider tinnitus biographical disruption (22, 60) and subsequent consequences on personal resources, that would remain otherwise unaddressed through standardized questionnaires.

Our understanding of the challenges encompassed in the tolerance of tinnitus (9) can also be extended by further considerations on *progressive* and *stable* narratives regarding tinnitus and the personal life story. Two of the five participants (namely Eva and Erik) have elaborated a rather distinct form of narrative on their self-experience with the presence of tinnitus. Considering that persistent tinnitus is a chronic condition, whose course may vary depending on the patient's general health status, it can be argued that an alternation of *progressive* and *stable* narratives is likely to occur during the lifespan. Tinnitus disappearance (i.e., suppression) remains to be an *unattainable* goal for most patients, but a *stable* form of narrative may reflect an individual's long-term relationship to the condition. Indeed, stability in self-experience toward valued goals and life orientation can be considered as accounting for the ability to remain unaffected by the losses and strains the narrator is being subjected to. A precautious appraisal of personal resources, along with required self-mastery toward disablement, also characterize stability in a self-narrative. Erik's experience is illustrative of a *stable* narrative in the face of a chronic condition such as tinnitus. Interestingly, Erik's narrative also integrates the *fulfillment* of basic psychological needs (58), with regard to his sense of competence (i.e., controlling intrusiveness of symptoms) and autonomy (i.e., being a pensioner, he can organize his day according to his own pace and needs). In addition, the need for relatedness is also satisfied in Erik's narrative, with a supportive spouse and a sense of belonging to the community of manufacture workers, whose lot is to cope with the inevitable physical byproducts of manual work. Therefore, not only does a *stable* narrative seem to be free from deprivation, which is characteristic of *regressive* narratives (see above), but it is also filled with several *resources* (i.e., social “nutriments”), according to Ryan and Deci (57). Thus, a *stable* narrative may reflect a balance that has been found by the sufferer, between strains and personal resources, serving to sustain sound self-confidence on a long-term basis.

In line with the conservation on the resources paradigm of stress (59), a *progressive* narrative can be considered to reflect *enhanced* vitality and orientation in life, as a result of self-enrichment and growth. A feature that may distinguish a *progressive* from a *stable* narrative is the role of self-knowledge that goes along with the strength of orientation in life. The ability to turn potential threats into challenges and to cope entails a capacity for reflection about oneself, and for sustaining engagement in subsequent adjustments to altered circumstances in life. Eva's narrative is illustrative of this form of self-presentation. *Progressive* narratives (56) also show similarities to the core construct of the salutogenesis model of health promotion, i.e., the *sense of coherence* (SOC) in the face of complexity and conflicts in a modern world. SOC has been defined as a global orientation toward life challenges that relies on the ability to find the world comprehensible, manageable, and meaningful (1). According to Antonovsky (62), it is mandatory for an individual to cope with accumulating challenges and factors that include risk of being overwhelmed by life circumstances when events cannot be translated into relevant information. Meaning is considered as a means to restore order from the risk of bewilderment facing the world's complexity and conflict. Thereby, SOC reflects the individual's ability to pursuit attainable goals, to consider manageable means, and sustain self-determination. *Progressive* narratives can be understood as the discursive enactment of SOC, with personal purposes and values in life that must be satisfied. Although referring to distinct concepts, the salutogenesis model (1, 62) and self-determination theory (57) have many features in common. Especially, both emphasize the role of *social* nutriments [e.g., social support, (57)] and *supra-structures* [i.e., health services, (62)] in maintaining order and orientation at an individual level of life experience. The tone of narration provides information over and above how meaning is made, the tone of narration provides information about whether the story is *progressive, stable* or *regressive*. They are collective constructs because they integrate forces and resources originating from *social bonds* in the broad sense of the term. Both Eva's (*progressive*) narrative and Erik's (*stable*) narrative have goals and values to pursuit, relying on others' attitudes and understanding toward their suffering.

The *regressive* narratives, in sharp contrast with the *progressive* narratives of our study, (Frida's, Ulla's and Karin's) are related to basic psychological deprivation of needs, i.e., the more suffering participants are left alone, lacking appropriate support from supra-structures they addressed (e.g., the lack of recognition Ulla receives from her insurance company regarding her tinnitus as a result of the car accident). Unfortunately, suffering patients with tinnitus often wander through an uncertain health care journey that fuels their distress (50). Here, two forms of supra-structures may be distinguished from one another, i.e., health services (professional perspective) and tinnitus support groups (lay perspective). Pryce et al. (29) recently reported that social connectedness and information sharing were two essential features of valuable resources found by suffering patients in support group settings. Improving patients' sense of coherence (i.e., manageable information) and fulfillment of basic psychological needs (i.e., relatedness) are both the kind of resources consistent with salutogenetic and self-determination perspectives on improved tolerance to tinnitus.

Interestingly, rumination (or ruminative self-focused thoughts) has often been associated with the pursuit of unattained goals (63) and the meeting with unattainable goals (64), as well as the lack of relatedness with others (58). All of these issues can be transposed to the nature of tinnitus, being an intrusive presence that cannot be removed from the individual's perception, which hinders the pursuit of everyday goals. Moreover, it can turn into a barrier to social relationships or occasionally also serve as an obstacle or an excuse for being socially involved (17). Conversely, it has long been recognized that *absorbing* pleasant and meaningful hobbies can bring momentary relief to patients from the intrusiveness of their tinnitus (65). These two directly opposed observations (i.e., rumination and full commitment) are consistent with a dynamic and optimal experience (the “flow” model) as an approach to intrinsic motivation (66, 67). Furthermore, it has been emphasized that full commitment excludes self-consciousness (and rumination) from an ongoing activity (68). As soon as self-consciousness emerges in the individual's perception, the optimal experience of being fully absorbed by an ongoing activity *ceases*. A sensitive dynamic process like this may have a substantial impact on patients' sense of ability to cope with tinnitus (9).

### Limitations and Strengths

The results of the present study should be considered in light of some limitations. One limitation is that the generalizability of the results is limited due to the small sample size and the choice of methodology. However, we would like to refer to the narrative psychological approach in which an important goal is to collect detailed, information-rich data (22), which also means that the number of participants is less crucial for the outcome. The material in the study consists of 15 interviews, of which most interviews are an hour long or sometimes even longer. We believe that it is correct to regard this material as information-rich data. However, our study results cannot be used for generalization purposes, and therefore we advise readers not to make uncritical generalizations from our results. Participants interviewed for the study were from one single health care system, which justifies the findings' lack of transferability across other health care settings. They were also mainly female and homogeneous in age, and therefore we cannot comment on gender or age differences. However, we have brought to the forefront those aspects of living with tinnitus that were perceived as important to the participants, while the aim was not to focus on recruiting a representative sample from which findings could be generalized.

The purpose of our study was to highlight the unique nature of personal experiences of having tinnitus, hoping that the narratives presented would contribute to showing a nuance of the complexity of the problem. Approaching the suffering of tinnitus as a *regressive* narrative enables the clinician to consider subsequent consequences of tinnitus on personal resources. In this approach, the consulting dialogue should focus on the patients' own comprehension of what the condition has implied for them psychologically and for their social belonging. From a rehabilitation perspective, a central issue is to help suffering patients to extend their ability for full commitment in life, while overcoming rumination about past events and unsolved struggles. This perspective can be framed as the passing from a *regressive* to a *progressive* (or even *stable*) narrative about living with tinnitus. When people are faced with challenges in life, such as illness or injury, these challenges interact with a unique individual carrying a history. The humanistic psychological view of what it implies to live with long-term chronic pain, tinnitus or other stress-related disorders with often multifaceted origins, is a complement to medical science with a preference for structured questionnaires and generic instruments. Perhaps though, in the future, narratives of the present study can be used for the construction of questionnaires by which more patients with tinnitus can be reached and this may contribute to new insights.

## Conclusion

To our knowledge, this is the first study which considers the daily experience of living with tinnitus in a broader perspective of the narration of individuals' life stories. The literature on tinnitus has remained rather symptom-focused, to the detriment of a more holistic approach to tinnitus (i.e., with concern for biographical backgrounds to the patients' suffering). This appears to be in sharp contrast with research on chronic illness [e.g., Bury (60)], chronic pain [e.g., Smith and Osborn (69)], and trauma studies [e.g., Crossley (22)], that all have emphasized the disruptive feature of chronic conditions with regard to everyday routines. A narrative perspective on tinnitus enlightens its potential impact on the sense of self, life orientation and experienced time, that are seldom addressed in the literature. Illness or injury, such as tinnitus, can have detrimental effects on a person's life and goals, sense of identity and social relationships, as illustrated by the participants' narratives in the present study.

An important goal of rehabilitation is to facilitate and strengthen the possibility of a life that is perceived as meaningful to the particular person by focusing on adaptive coping skills, enhanced resilience, improved functioning and control in areas of life where mastery is still possible and necessary for realizing personal life goals. To meet a suffering patient is challenging because it demands a special interest for the person as a complex, living system. Hall underlines the importance of accuracy in the way clinicians perceive their patients, and she outlines the principles of the concept of *interpersonal sensitivity*. Further, she describes a number of states and traits that clinicians should be aware of in communication with their patients, for example feelings, desires, truthfulness, intentions, needs, physical states, attitudes, beliefs, and values (70). Hall argues that in the area of health communication, only the behavioral aspect (saying or doing, or not saying or doing) is studied, while the perceiving aspect (noticing, interpreting) has been ignored. Finally, we want to acknowledge the clinician's own horizon of experience to be used as a guide to the inner life of our patients (71). The ability to be empathic includes the capacity to interpret non-verbal language, mimics, gestures, and body posture—skills that should be optimal in clinical practice. There is no direct entrance to the patient's inner experience even if we try to understand what it means to feel that one's entire existence is circumscribed by the presence of tinnitus. It is only through our conception or imagination that we can attempt to grasp what the other person is experiencing.

## Data Availability Statement

All datasets generated for this study are included in the article/Supplementary Material.

## Ethics Statement

The studies involving human participants were reviewed and approved by The Ethical Committee of Gothenburg. The patients/participants provided their written informed consent to participate in this study.

## Author Contributions

SE, LL, and ND planned the study and the design together. SE was responsible for ethical application and the contacts with the Swedish hospital staff for patient involvements in the study. LL and SE made contacts with the participating patients prior to the study. Interviews and transcriptions were undertaken by SE and LL. A major part of the transcripts were translated to ND (Not Swedish spoken) by SE. All three researchers, SE, LL, and ND took part in the narrative analyses and provided expertise, research experience, literature, and references. SE, LL, and ND were also all active in writing and the manuscript took form by the involvement of all researchers.

### Conflict of Interest

The authors declare that the research was conducted in the absence of any commercial or financial relationships that could be construed as a potential conflict of interest.
