# Supplementary material for: The Experience of Tinnitus and Its Interaction With Unique Life Histories—Life Events, Trauma and Inner Resources Narrated by Patients With Tinnitus
Source: Front Psychiatry. 2020 Mar 18;11:136. doi: 10.3389/fpsyt.2020.00136 (PMC7093576; doi:10.3389/fpsyt.2020.00136)
Supplement: Supplementary file 1 [file Data_Sheet_1.docx]

**APPENDIX**

**Excerpts illustrating themes in the narratives of the five particpants.**

**Karin**

*I: You have, as you said, headaches. Do you know why?*

*P: Both yes and no. I consulted a specialist regarding my headaches for the first time 30 years ago. I have exchanged all amalgam in my teeth. They made an X-ray of the skull; I believe two times. I got two relaxation courses and they believed that the headaches are linked to my back.*

*******

*I: Does tinnitus effect your sleep?*

*P: I wake up with headache and vertigo and keep hearing this (tinnitus) the whole time. It may happen after some extreme and intensive days. Yes, it has an effect on my sleep and it is a stressor, when you know, like, I plan to go somewhere and there will be a lot of people. Can I manage that?*

*******

*I: Is tinnitus a problem when you are with people or does it influence your social life?*

*P: When we are in a meeting … it is a big trouble. This might seem mean, but some people have a vocal pitch that triggers my tinnitus. I don’t believe they can help it, and they can be very kind people … Then I have to sit nicely with my hand over the ear. It simply does not work – some people have a vocal pitch that doesn’t correspond with my hearing.*

*******

*I: Do you experience that tinnitus has had an effect on quality of life up to now, or is that a too strong word perhaps?*

*P: Both yes and no. I suffered from burnout 14 years ago (the voice becomes tense).*

*I: Yes…*

*P: …. ended up in a depression. And it took more than three years to come back. It changed one’s way of thinking very much, today you play down certain things instead. Tinnitus is behind the frustration – can’t it be quiet?*

*I: You don’t become depressed?*

*P: No, because I believe that I am afraid to end up in that situation again and this is when I take my medicine: “work in the garden”. The headache disappears and the surrounding sounds make me think less about this (tinnitus).*

*******

*I: You mean you don’t get stuck in a way of thinking, instead you are trying.. (here interrupted by P)*

*P: Yes…. this time I can ask for help - something I couldn’t before.*

*___________________________________________________________________________*

**Ulla**

*I: Can you tell me what happened, why did you have tinnitus, please tell me about that.*

*P: It came quickly. I was driving my cab in the city center, and then a car came in full speed from left, straight out on the road, and there was not a chance for me to brake or to stop my car”…The car ended up on a refuge, but I was able to turn the steering-wheel around, otherwise it had ended up in the ditch nearby a sand-bank. I went out of the car and looked and saw the crash and then I felt a terrible ache in my head and neck…..Later that evening the doctor said that he wanted me to stay at the hospital, because you had a terrible blow to your head he said. I rather wanted to go home and noticed directly when coming home that this iiiiiiiiiiiiiii, like this, the ringing in the ears and thought that it was a side-effect, so it might stop, but it didn’t…. instead it has continued.*

*******

*I: Did you report the accident to the insurance company?*

*P: “Yes, I did, directly after the accident and then again and again, but nothing happened, so I got tired of it, and contacted a lawyer.”*

*******

*I: Can I ask you – what effects these worries had on you?*

*P: It varies very much, I can say, on one hand I am still angry about losing my car, my working life has been destroyed by all this. It somehow still lingers with me. …. But it is also that it goes idle to have this constant ringing in the ears and it bothers me most when I lay on the left side at night….*

*I: Has tinnitus been the same since it started?*

*P: Yes, it has.*

*I: The character hasn’t changed?*

*P: No, it hasn’t. And then, like when I am driving the cab, I don’t notice it so much.*

*I: I understand, because there is so much noise.*

*P: There are so many different sounds, it is the buzz from the wheels and people sit by my side and narrate their life story – about good doctors and stupid doctors and medical travels (laughing) illnesses and childhood and everything (laughing) - talking continuously.*

*******

*P: I don’t know how to come further with this – if I knock on the health center door in town nothing happens, they don’t even respond when they have the answers from my examinations.*

*I: No, it’s not so good.*

*___________________________________________________________________________*

**Eva**

*I: Can you describe your experiences with tinnitus since tinnitus started ten years ago?*

*P: I notice that I am tense for some reason, and my sleep has been worse as well as other things around me…. When I am having a good and enjoyable period in my life, I am more relaxed. I am less distressed but it (tinnitus) is there all the time.*

*******

*I: If you should wish for help to handle tinnitus what would that be?*

*P: Yes, the help I received from the Hearing Center should have come earlier, much, much earlier. It was very confirming. I got practical and concrete tips and advice on how to think about it. Before that I got in contact with a dental hygienist and talked to her. I understood that I clench the jaw a lot … and I got a splint, which I have been trying to use.*

*I: Does it help?*

*P: Yes, I don’t know if it is a help for tinnitus, but it makes my jaws less stiff… I think that my sleep is better when I wear the splint.*

*******

*I: Did you know about tinnitus before you yourself got tinnitus?*

*P: Yes, I did.*

*I: But your mother – she didn’t talk about it?*

*P: No, she didn’t say anything. It was first when I complained about it sitting next to my brother and we found out that we had the same problem she (the mother) suddenly said: Yes, I have this too. But it is nothing to complain about (interviewee laughing).*

*******

*I: Have you met other people with tinnitus and exchanged thoughts with them?*

*P: Yes, I probably have. I don’t know if there is a tinnitus association, I have not joined any… I read about tinnitus in articles and think I should do more, really try relaxation and perhaps massage and yoga…outdoor life being in the open is clearly the best therapy. Without doubt it has saved and helped me to overcome my crises. I can be very sad and tired but after half an hour in the woods I notice that the corners of my mouth have lifted.*

*I: Is this something you acquired when growing up?*

*P: Yes, absolutely. I grew up in the countryside and my dad loved the outdoor life and so do all of us siblings.*

*___________________________________________________________________________*

**Frida**

*I: You have described that certain situations make tinnitus worse, such as, for example a stressful job situation. Are there other situations that contribute to making tinnitus worse?*

*P: I used to be much more social. I lead a much quieter life today since going to the pub and other social events is no fun anymore as I cannot hear. When I do not hear I feel bad (she starts to cry). When I feel bad, I do not function. My soul hurts.*

*******

*I: How would you describe your upbringing?*

*P: As very tough. The reason why we moved to another country was that I was raped by a friend of my fathers. It did not happen once. My mother wanted to find a safe place for me and the rest of the family. I have carried around feelings of guilt as I was the reason for the move.*

*******

*I: You mentioned that you have had panic attacks.*

*P: Yes, I developed panic attacks after my husband died.*

*I: Do you see a connection between panic attacks and tinnitus?*

*P: If I have a panic attack tinnitus often increases.*

*******

*I: Do you have many friends?*

*P: I do not have many people around me anymore, they all disappeared after my husband died. They were his friends.*

*I: You have mentioned that you find it difficult to build a social network. What, in particular, do you find difficult?*

*P: Tinnitus bothers me very much. The more I have learned about it the more I have realized how much I have isolated myself.*

*__________________________________________________________________________*

***Erik***

*I: Do you mean that you have seen a therapist in order to receive help accepting this? I mean physical illness and tinnitus.*

*P: Yes, in order to deal with losing my job, coming to terms with my illness, to be able to accept. During this time, I learned a lot about positive thinking, which has helped me tremendously.*

*******

*I: my next question is whether you experience support from family, relatives and friends?*

*P: Yes, absolutely, possibly because my relatives have a background in working class and heavy industry. Tinnitus is well known, several of my relatives have had tinnitus. My dad had tinnitus, he worked as a welder, so no problem.*

*******

*I: You mention that you as a child spoke to your parents, or to your dad about tinnitus?*

*P: Yes, my dad has had tinnitus for as long as I can remember…*

*I: How did he react when you told him about your tinnitus?*

*P: It was nothing to worry about, nothing dangerous, kind of.*

*I: So, you did not feel very worried then?*

*P: No, no, not at all.*

*******

*I: Has your attitude to life changed?*

*P: No, I don’t know. I was active in politics before I got ill. Then, my children were born, and I prioritized them. But thoughts about politics are with me, still. My frustration today is that I have not enough strength to get the message out and act. I write one or another letter to the editor hoping that I somehow…. in any case can do something (laughing).*
